# Assessment of Lectin Staining Biomarkers using a Murine Model of GNE Myopathy

**DOI:** 10.17912/micropub.biology.001717

**Published:** 2025-10-08

**Authors:** Olivia Parker, Jordyn Woods, Max Rothkopf, Daniel Drach, Hanna N. Wetzel, Kelly E. Crowe

**Affiliations:** 1 Biology, Xavier University, Cincinnati, Ohio, United States

## Abstract

GNE myopathy (GNEM) is a rare myopathy caused by mutations in the UDP-GlcNAc epimerase/ManNAc-6 kinase *(GNE)* gene, which reduce sialic acid (SA) biosynthesis and impair muscle through unclear mechanisms. As development of SA-restoring GNEM gene therapies is underway, it is essential to develop SA-detecting biomarkers in preclinically-relevant murine tissues. Here, we assess skeletal muscle staining of the *Gne ^M743T/M743T^* GNEM model with four sialylation-detecting lectins. While no tested lectins could effectively differentiate between *Gne ^M743T/M743T^* and wild type tissues, Peanut Agglutinin (PNA) showed differential binding in tissues with and without SA-removing sialidase treatment, indicating its promise in detecting hyposialylation in murine tissues.

**Figure 1. Lectin Staining in Murine Models of GNEM:**
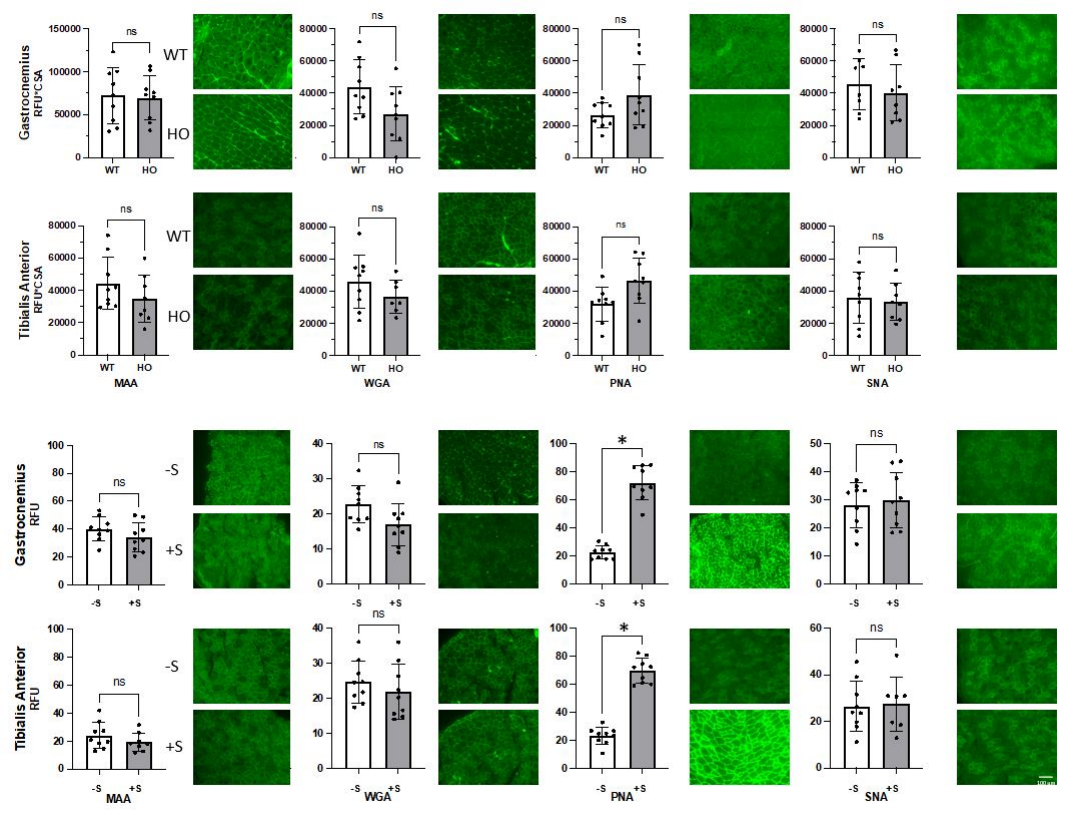
Lectin (MAA, WGA, PNA, and SNA) staining in homozygous wild type (WT) and *Gne ^M743T/M743T^ (HO)* (top), and WT with and without sialidase treatment (–S and +S, respectively) (bottom) in gastrocnemius and tibialis anterior skeletal muscle. Bar graphs represent mean±SD of RFU*CSA for WT/HO, and RFU for –S/+S plots. Significance (denoted with asterisks) was determined using t-tests followed by a false-discovery correction. Representative images are shown beside their respective bar graphs. Scale bar represents 100μm.

## Description

GNE myopathy (GNEM) is a rare autosomal myopathy with increased prevalence in Japanese and Iranian Jewish populations due to the presence of founder mutations. Symptoms typically begin in the third decade of life with distal weakness and slow proximal progression, eventually leading to loss of ambulation (Argov & Mitrani Rosenbaum, 2015).

GNEM is caused by mutations in the Glucosamine (UDP-N-Acetyl)-2-Epimerase/N-Acetylmannosamine Kinase *(GNE)* gene, which encodes a bifunctional enzyme that catalyzes steps of the sialic acid (SA) biosynthetic pathway. As such, SA abundance is generally reduced in GNEM patient tissues, leading to skeletal muscle pathology through an unclear mechanism. SA is a negatively charged, terminal glycan of the glycocalyx, modulating diverse aspects of skeletal muscle physiology such as gating of voltage-gated ion channels, myogenesis, and oxidative stress (Champigny et al., 2005; Cho et al., 2017; Johnson et al., 2004; Schmitt et al., 2022; Schwetz et al., 2011).

Gene therapy to treat GNEM seeks to provide a corrected copy of the *GNE* gene though a vector such as adeno-associated virus (AAV) (Mitrani-Rosenbaum et al., 2012, 2022; Nemunaitis et al., 2010, 2011). To support the development of these therapies, it is important to develop Investigative New Drug (IND)-enabling preclinical biomarkers using mouse models such as the *Gne ^M743T/M743T^* model, which was reported to show muscle hyposialylation and has been used for therapeutic development for oral glycan therapies (Fleming & Powers, 2012; Lochmüller et al., 2019; Niethamer et al., 2012; Xu et al., 2017). An ideal biomarker for gene therapy would be staining-based to allow visualization of a transgene or its product for quantification of transduction efficiency (Hakim et al., 2020).

Lectins, linkage-specific carbohydrate-binding proteins, could act as such a preclinical staining biomarker by measuring sialylation in skeletal muscle (Leoyklang et al., 2018; Sharon, 2007; Tajima et al., 2005). To act as a viable biomarker, lectins that directly bind SA would show reduced binding in GNEM, while lectins that bind underlying sugar structures that are unmasked in SA’s absence would show increased binding in GNEM (Leoyklang et al., 2018; Saito et al., 2004; Tajima et al., 2005; Voermans et al., 2010). Several lectins have shown altered binding in skeletal muscle cells due to SA alterations, including *Sambucus nigra* agglutinin (SNA), Wheat germ agglutinin (WGA), *Maackia amurensis* agglutinin (MAA), and Peanut Agglutinin (PNA) (Leoyklang et al., 2014, 2018; Niethamer et al., 2012; Noguchi et al., 2004; Zhang et al., 2018; Zygmunt et al., 2023).

It is critical to have a robust measure of hyposialylation due to its importance as a preclinical gene therapy outcome metric in *Gne ^M743T/M743T^* model. Although various lectins have been shown to reflect sialylation in various *in vitro* models, murine models, and GNEM patient tissue samples, they have not been directly compared in their ability to assess sialylation in a preclinical murine model. Here, we assess a panel of SA-detecting lectins for their efficacy in SA detection at approximately 1 and 2 months of age, common gene therapy injection timepoints (Gray, 2016). We use both the *Gne ^M743T/M743T^* GNEM murine model specifically and wild-type (WT) muscles with and without enzymatic SA removal to assess these lectins in murine tissues more generally, allowing identification of lectins that could act as IND-enabling, preclinical biomarkers and potentially translate into outcome measures for a GNEM gene therapy clinical trial.

We first compared lectin staining in *Gne ^M743T/M743T^* and WT gastrocnemius and tibialis anterior (TA) muscles using sialylation-detecting lectins *Maackia Amurensis* agglutinin (MAA), peanut agglutinin (PNA), *Sambucus Nigra* agglutinin (SNA), and wheat germ agglutinin (WGA). In all cases, we found no significant differences between *Gne ^M743T/M743T^* and WT skeletal muscle samples after correcting for multiple comparisons, though PNA staining in TA was significantly different prior to application of the false discovery correction (p=0.0217).

We next sought to enzymatically treat WT skeletal muscle with the SA-removing sialidase enzyme (Minami et al., 2021) to assess the ability of each lectin to detect sialylation changes in murine tissues more generally *.* Here, we found that PNA binding in WT mouse muscle showed a statistically significant increase after sialidase treatment, with a 3.2-fold increase (p<0.0001) in gastrocnemius and a 3.0-fold increase (p<0.0001) in the TA. MAA, SNA, and WGA did not show a statistically significant change between sialidase-treated and -untreated WT skeletal muscle in either the gastrocnemius or TA after correcting for multiple comparisons, though prior to application of the false discovery correction, WGA staining in gastrocnemius was significantly lower in sialidase-treated muscle (p=0.0479).

Overall, this study used a series of lectins to stain skeletal muscle of a GNEM mouse model. In addition to assessing differences between WT and *Gne ^M743T/M743T^* mice, we also evaluated the utility of these lectins in murine models more generally by comparing lectin staining with and without sialidase, which cleaves SA residues from underlying glycans (Minami et al., 2021). The panel of lectins investigated herein were chosen from the literature based on their prior use in quantifying hyposialylation in biopsies of patients with GNEM (Leoyklang et al., 2014, 2018; Saito et al., 2004; Voermans et al., 2010). To act as a useful biomarker, SA-binding lectins (such as MAA, SNA, and WGA) would show reduced binding in *Gne ^M743T/M743T^* or sialidase-treated WT tissues, while lectins that bind sugar structures underlying SA (such as PNA) would show increased binding in *Gne ^M743T/M743T^* or sialidase-treated WT tissues (Leoyklang et al., 2018; Saito et al., 2004; Tajima et al., 2005; Voermans et al., 2010).

First, we demonstrated that none of the four lectins tested (WGA, PNA, SNA, MAA) showed differential binding in WT vs *Gne ^M743T/M743T^* skeletal muscle. This is in contrast to previous studies in human biopsies from patients with GNEM, which have found that SNA (Leoyklang et al., 2014, 2018; Saito et al., 2004), WGA (Broccolini et al., 2008; Saito et al., 2004), and MAA (Saito et al., 2004) decrease in biopsies from patients with GNEM as compared to non-GNEM patients via either lectin staining or lectin blotting, although other studies report no change (Leoyklang et al., 2014; Nemunaitis et al., 2010; Noguchi et al., 2004; Tajima et al., 2005; Voermans et al., 2010). This inconsistency may be due to mutation-specific variability in hyposialylation, with sialylation being relatively well-preserved in the p.M743T mutation as compared to other common mutations (Celeste et al., 2014). Additionally, previous studies have shown that SNA binding is decreased in the Gne ^M743T/M743T^ mouse model of GNEM, though they use formalin-fixed tissues, as compared to fresh-frozen tissues used herein (Niethamer et al., 2012). Also in contrast to our results in the Gne ^M743T/M743T^ model, PNA binding has been shown to increase in patients with GNEM (Saito et al., 2004; Tajima et al., 2005; Voermans et al., 2010). Of note, in our results, PNA staining was significantly higher prior to application of a false discovery correction, so it is possible that with a larger sample size we would be able to detect differences between these groups using this lectin. In addition, one limitation of this work is that we do not address the extent to which muscle characteristics such as fiber type distribution may affect these results. Overall, it is possible that these lectins behave differently in this mouse model than in human biopsies, which raises concerns for the future use of this model in pre-clinical drug-development work.

Based on these results, we next tested the ability of these lectins to meaningfully detect changes in SA levels in WT murine tissues using sialidase, an enzyme that reliably removes SA from underlying glycans (Minami et al., 2021). In our study, PNA was the only lectin that showed a significant difference between sialidase-treated and -untreated muscle after correction for multiple comparisons, though WGA was significant before correction. This robust decrease in PNA binding is consistent with work in human biopsies that have observed lower PNA staining in GNEM patient tissue (Saito et al., 2004; Tajima et al., 2005; Voermans et al., 2010). The lack of significant difference in binding of WGA, SNA, and MAA would indicate that these lectins are not a reliable measure of SA in mice. These findings highlight the importance of further evaluation of lectin staining validity in murine tissues.

In summary, our findings indicate that PNA has the most promise in assessing altered sialylation in murine tissues as compared to WGA, SNA, and MAA. In addition, these findings would indicate that caution should be exercised in utilizing lectin staining as an outcome measure for preclinical trials of sialylation-restoring GNEM therapies in *Gne ^M743T/M743T^* skeletal muscle.

## Methods

**Mice**

Skeletal muscle tissues were obtained from wild type (WT) (n=9, 3 males and 6 females) and homozygous *Gne ^M743T/M743T^* (HO) (n=9, 5 males and 4 females) mice that were housed and euthanized at Charles River Laboratories and cared for under the Animal Care and Use Committee of Charles River Laboratories Canada. Animals were sacrificed at 4-10 weeks.

**Lectin Immunostaining**

Gastrocnemius and tibialis anterior muscles were flash frozen and sectioned at 10µm using a cryostat. For lectin staining, where indicated, samples were incubated in PBS (-S) or treated with 0.3 units/mL neuraminidase (sialidase) from *Clostridium perfringens* ( *C. welchii* ) (+S) for 30 minutes at 37°C and washed in PBS. Next, sections were blocked in 10% goat serum for one hour, then incubated with biotinylated *Maackia Amurensis* agglutinin (MAA), fluorescein-conjugated peanut agglutinin (PNA), fluorescein-conjugated *Sambucus Nigra* agglutinin (SNA), or fluorescein-conjugated wheat germ agglutinin (WGA) for one hour. For MAA staining, sections were washed with phosphate-buffered saline (PBS) and incubated with FITC-Streptavidin for one hour. All sections were washed in PBS and then mounted with ProLong Gold Antifade Mountant with DAPI. Exposure time-matched images were acquired for each lectin at 20X using a Z-X800E Keyence Fluorescence Microscope, with 2-4 images analyzed per sample.

**Quantification and Statistical Analysis**

Relative fluorescence was quantified in each image using ImageJ, then normalized to average fiber diameter within each mouse (quantified via Cellpose as described elsewhere (Stringer et al., 2021)) via multiplication by cross-sectional area (CSA). Determinations of significance between the two groups were assessed using an unpaired two-tailed Students’ t test; statistics were performed using Prism software version 9.3.0 (GraphPad; San Diego, CA) with a false-discovery correction to account for multiple comparisons (Curran-Everett, 2000).

## Reagents

**Table:** 

Lectin	Glycan Specificity	Available From
*Maackia amurensis* agglutinin (MAA)	Siaα2-3Gal	Vector Laboratories
Peanut Agglutinin (PNA)	Galβ1-3GalNAcα1-Ser/Thr	Vector Laboratories
*Sambucus nigra* agglutinin (SNA)	Siaα2-6Gal/GalNAc	Vector Laboratories
Wheat germ agglutinin (WGA)	Sia, GlcNAc(β1,4)GlcNAc	Vector Laboratories

**Table:** 

Animal	Stain/Genetic Background	Obtained
WT	C57BL/6J	Charles River Laboratories
* Gne ^M743T/M743T^*	C57BL/6J with M743T mutation in the *Gne* gene	Charles River Laboratories

## References

[R1] Argov Zohar, Mitrani Rosenbaum Stella (2015). GNE Myopathy: Two Clusters with History and Several Founder Mutations. Journal of Neuromuscular Diseases.

[R2] Broccolini Aldobrando, Gidaro Teresa, De Cristofaro Raimondo, Morosetti Roberta, Gliubizzi Carla, Ricci Enzo, Tonali Pietro A., Mirabella Massimiliano (2007). Hyposialylation of neprilysin possibly affects its expression and enzymatic activity in hereditary inclusion‐body myopathy muscle. Journal of Neurochemistry.

[R3] Celeste Frank V., Vilboux Thierry, Ciccone Carla, de Dios John Karl, Malicdan May Christine V., Leoyklang Petcharat, McKew John C., Gahl William A., Carrillo-Carrasco Nuria, Huizing Marjan (2014). Mutation Update for
*GNE*
Gene Variants Associated with GNE Myopathy. Human Mutation.

[R4] Champigny Marc J., Perry Robert, Rudnicki Michael, Igdoura Suleiman A. (2005). Overexpression of MyoD-inducible lysosomal sialidase (neu1) inhibits myogenesis in C2C12 cells. Experimental Cell Research.

[R5] Cho Anna, Christine May, Malicdan V., Miyakawa Miho, Nonaka Ikuya, Nishino Ichizo, Noguchi Satoru (2017). Sialic acid deficiency is associated with oxidative stress leading to muscle atrophy and weakness in GNE myopathy. Human Molecular Genetics.

[R6] Curran-Everett Douglas (2000). Multiple comparisons: philosophies and illustrations. American Journal of Physiology-Regulatory, Integrative and Comparative Physiology.

[R7] Fleming Thomas R., Powers John H. (2012). Biomarkers and surrogate endpoints in clinical trials. Statistics in Medicine.

[R8] Gray Steven J (2016). Timing of Gene Therapy Interventions: The Earlier, the Better. Molecular Therapy.

[R9] Hakim Chady H., Clément Nathalie, Wasala Lakmini P., Yang Hsiao T., Yue Yongping, Zhang Keqing, Kodippili Kasun, Adamson-Small Laura, Pan Xiufang, Schneider Joel S., Yang N. Nora, Chamberlain Jeffrey S., Byrne Barry J., Duan Dongsheng (2020). Micro-dystrophin AAV Vectors Made by Transient Transfection and Herpesvirus System Are Equally Potent in Treating mdx Mouse Muscle Disease. Molecular Therapy - Methods & Clinical Development.

[R10] Johnson Daniel, Montpetit Marty L., Stocker Patrick J., Bennett Eric S. (2004). The Sialic Acid Component of the β1 Subunit Modulates Voltage-gated Sodium Channel Function. Journal of Biological Chemistry.

[R11] Leoyklang Petcharat, Class Bradley, Noguchi Satoru, Gahl William A, Carrillo Nuria, Nishino Ichizo, Huizing Marjan, Malicdan May Christine (2018). Quantification of lectin fluorescence in GNE myopathy muscle biopsies. Muscle & Nerve.

[R12] Leoyklang Petcharat, Malicdan May Christine, Yardeni Tal, Celeste Frank, Ciccone Carla, Li Xueli, Jiang Rong, Gahl William A, Carrillo-Carrasco Nuria, He Miao, Huizing Marjan (2014). Sialylation of Thomsen–Friedenreich Antigen is a Noninvasive Blood-Based Biomarker for GNE Myopathy. Biomarkers in Medicine.

[R13] Lochmüller Hanns, Behin Anthony, Caraco Yoseph, Lau Heather, Mirabella Massimiliano, Tournev Ivailo, Tarnopolsky Mark, Pogoryelova Oksana, Woods Catherine, Lai Alexander, Shah Jinay, Koutsoukos Tony, Skrinar Alison, Mansbach Hank, Kakkis Emil, Mozaffar Tahseen (2019). A phase 3 randomized study evaluating sialic acid extended-release for GNE myopathy. Neurology.

[R14] Minami Akira, Kurebayashi Yuuki, Takahashi Tadanobu, Otsubo Tadamune, Ikeda Kiyoshi, Suzuki Takashi (2021). The Function of Sialidase Revealed by Sialidase Activity Imaging Probe. International Journal of Molecular Sciences.

[R15] Mitrani-Rosenbaum Stella, Yakovlev Lena, Becker Cohen Michal, Argov Zohar, Fellig Yakov, Harazi Avi (2022). Pre Clinical Assessment of AAVrh74.MCK.GNE Viral Vector Therapeutic Potential: Robust Activity Despite Lack of Consistent Animal Model for GNE Myopathy. Journal of Neuromuscular Diseases.

[R16] Mitrani-Rosenbaum Stella, Yakovlev Lena, Becker Cohen Michal, Telem Michal, Elbaz Moran, Yanay Nurit, Yotvat Hagit, Ben Shlomo Uri, Harazi Avi, Fellig Yakov, Argov Zohar, Sela Ilan (2012). Sustained expression and safety of human GNE in normal mice after gene transfer based on AAV8 systemic delivery. Neuromuscular Disorders.

[R17] Nemunaitis Gregory, Maples Phillip B., Jay Chris, Gahl William A., Huizing Marjan, Poling Justin, Tong Alex W., Phadke Anagha P., Pappen Beena O., Bedell Cynthia, Templeton Nancy S., Kuhn Joseph, Senzer Neil, Nemunaitis John (2010). Hereditary inclusion body myopathy: single patient response to
*GNE*
gene Lipoplex therapy. The Journal of Gene Medicine.

[R18] Nemunaitis Gregory, Jay Chris M., Maples Phillip B., Gahl William A., Huizing Marjan, Yardeni Tal, Tong Alex W., Phadke Anagha P., Pappen Beena O., Bedell Cynthia, Allen Henry, Hernandez Cathy, Templeton Nancy S., Kuhn Joseph, Senzer Neil, Nemunaitis John (2011). Hereditary Inclusion Body Myopathy: Single Patient Response to Intravenous Dosing of
*GNE*
Gene Lipoplex. Human Gene Therapy.

[R19] Niethamer Terren K., Yardeni Tal, Leoyklang Petcharat, Ciccone Carla, Astiz-Martinez Adrian, Jacobs Katherine, Dorward Heidi M., Zerfas Patricia M., Gahl William A., Huizing Marjan (2012). Oral monosaccharide therapies to reverse renal and muscle hyposialylation in a mouse model of GNE myopathy. Molecular Genetics and Metabolism.

[R20] Noguchi Satoru, Keira Yoko, Murayama Kumiko, Ogawa Megumu, Fujita Masako, Kawahara Genri, Oya Yasushi, Imazawa Masaoki, Goto Yu-ichi, Hayashi Yukiko K., Nonaka Ikuya, Nishino Ichizo (2004). Reduction of UDP-N-acetylglucosamine 2-Epimerase/N-Acetylmannosamine Kinase Activity and Sialylation in Distal Myopathy with Rimmed Vacuoles. Journal of Biological Chemistry.

[R21] Saito F., Tomimitsu H., Arai K., Nakai S., Kanda T., Shimizu T., Mizusawa H., Matsumura K. (2004). A Japanese patient with distal myopathy with rimmed vacuoles: missense mutations in the epimerase domain of the UDP-N-acetylglucosamine 2-epimerase/N-acetylmannosamine kinase (GNE) gene accompanied by hyposialylation of skeletal muscle glycoproteins. Neuromuscular Disorders.

[R22] Schmitt Rebecca E., Smith Douglas Y., Cho Dong Seong, Kirkeby Lindsey A., Resch Zachary T., Liewluck Teerin, Niu Zhiyv, Milone Margherita, Doles Jason D. (2022). Myogenesis defects in a patient-derived iPSC model of hereditary GNE myopathy. npj Regenerative Medicine.

[R23] Schwetz Tara A., Norring Sarah A., Ednie Andrew R., Bennett Eric S. (2011). Sialic Acids Attached to O-Glycans Modulate Voltage-gated Potassium Channel Gating. Journal of Biological Chemistry.

[R24] Sharon Nathan (2007). Lectins: Carbohydrate-specific Reagents and Biological Recognition Molecules. Journal of Biological Chemistry.

[R25] Stringer Carsen, Wang Tim, Michaelos Michalis, Pachitariu Marius (2020). Cellpose: a generalist algorithm for cellular segmentation. Nature Methods.

[R26] Tajima Youichi, Uyama Eiichiro, Go Shinji, Sato Chihiro, Tao Nodoka, Kotani Masaharu, Hino Hirotake, Suzuki Akemi, Sanai Yutaka, Kitajima Ken, Sakuraba Hitoshi (2005). Distal Myopathy with Rimmed Vacuoles. The American Journal of Pathology.

[R27] Voermans NC, Guillard M, Doedee R, Lammens M, Huizing M, Padberg GW, et al., Lefeber DJ. 2010. Clinical features, lectin staining, and a novel GNE frameshift mutatio. Clinical neuropathology. 29: 71-77. 371. PMC3500779 20175955

[R28] Xu Xin, Wang Amy Q., Latham Lea L., Celeste Frank, Ciccone Carla, Malicdan May Christine, Goldspiel Barry, Terse Pramod, Cradock James, Yang Nora, Yorke Selwyn, McKew John C., Gahl William A., Huizing Marjan, Carrillo Nuria (2017). Safety, pharmacokinetics and sialic acid production after oral administration of N -acetylmannosamine (ManNAc) to subjects with GNE myopathy. Molecular Genetics and Metabolism.

[R29] Zhang Xiaoqing, Nie Huan, Whited Joshua, Wang Dan, Li Yu, Sun Xue-Long (2018). Recent approaches for directly profiling cell surface sialoform. Glycobiology.

[R30] Zygmunt Deborah A., Lam Patricia, Ashbrook Anna, Koczwara Katherine, Lek Angela, Lek Monkol, Martin Paul T. (2023). Development of Assays to Measure
*GNE*
Gene Potency and Gene Replacement in Skeletal Muscle. Journal of Neuromuscular Diseases.

